# Impact of transcatheter edge-to-edge mitral valve repair on central sleep apnoea

**DOI:** 10.1007/s00392-022-02139-3

**Published:** 2022-12-12

**Authors:** Michael G. Paulus, Tobias Liedtke, Michael Hamerle, Christian Schach, Lars S. Maier, Stefan Stadler, Christoph Birner, Kurt Debl, Michael Arzt, Bernhard Unsöld, Christine Meindl

**Affiliations:** 1 Department of Internal Medicine II, University Hospital Regensburg, Franz-Josef-Strauß-Allee 11, 93053 Regensburg, Germany; 2 Department of Internal Medicine I, Klinikum St. Marien, Amberg, Germany

**Keywords:** Sleep apnoea, Sleep-disordered breathing, Cheyne–Stokes respiration, Mitral regurgitation, Transcatheter mitral valve repair, Edge-to-edge mitral valve repair

## Abstract

**Aims:**

Sleep-disordered breathing (SDB) and its subtype central sleep apnoea (CSA) are highly prevalent in patients with heart failure and associated with worse prognosis. Whereas pharmacological therapy of heart failure has been shown to ameliorate CSA, results from previous studies on the effect of mitral regurgitation therapy on SDB are contradicting. The aim of this study was to assess the impact of transcatheter edge-to-edge mitral valve repair (TEER) on prevalence and severity of CSA.

**Methods and results:**

We enrolled 47 patients undergoing TEER for symptomatic mitral regurgitation in a prospective study. Secondary mitral regurgitation and left ventricular ejection fraction < 50% were present in 79% and 68% of patients, respectively. Respiratory polygraphy was performed before TEER in a compensated condition and four weeks after the procedure. 34 patients completed the follow-up. At baseline, 19 (56%) patients showed moderate-to-severe SDB, of whom 13 (68%) were classified as CSA. Both apnoea-hypopnoea index and percentage of recorded time spent in Cheyne-Stokes respiration strongly decreased from baseline to follow-up (median [IQR] 16 [7–30] vs. 7 [4–15] /h, *p* = 0.007; 6 [0–34] vs. 0 [0–8] %, *p* = 0.008). Median relative reduction of central apnoea index was 75% (*p* = 0.023), while obstructive apnoea index did not change significantly. Increase in stroke volume after TEER and high systolic pulmonary artery pressure at baseline predicted a > 50% reduction of both Apnoea-hypopnoea index and Cheyne-Stokes respiration.

**Conclusion:**

TEER is associated with a significant short-term reduction of CSA and Cheyne-Stokes respiration in high-risk patients, strengthening its value as an effective treatment option for advanced heart failure.

**Graphical abstract:**

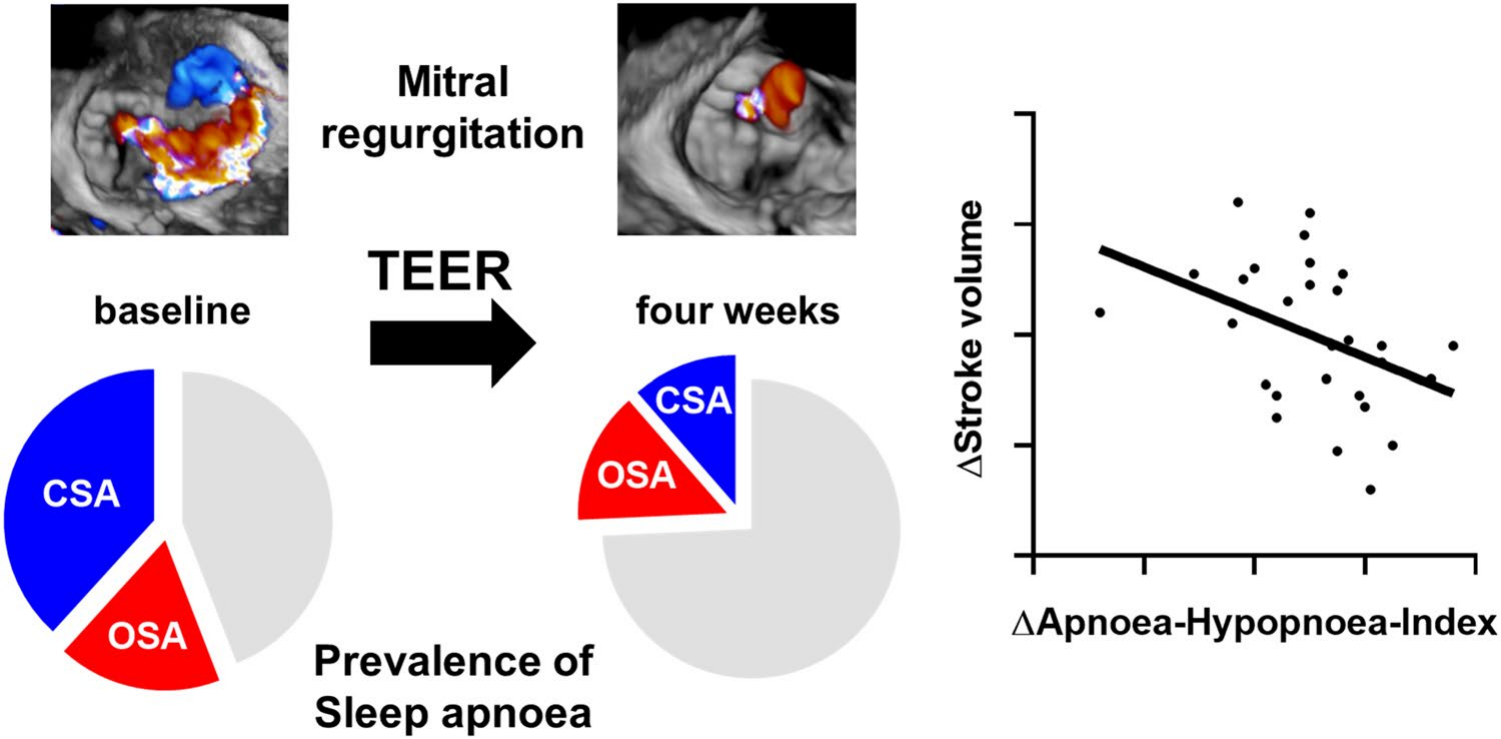

**Supplementary Information:**

The online version contains supplementary material available at 10.1007/s00392-022-02139-3.

## Introduction

Sleep-disordered breathing (SDB) is highly prevalent in the population suffering from chronic heart failure (HF), affecting approximately half of the patients with systolic dysfunction [1]. Both obstructive sleep apnoea (OSA) and central sleep apnoea (CSA) occur more frequently in HF and are independently associated with worse prognosis [2]. In particular, the presence of significant periodic Cheyne–Stokes respiration (CSR) may identify patients at high risk for cardiovascular death [3]. Whereas specific treatment of OSA may be beneficial in HF, management of CSA primarily focuses on the therapy of the underlying condition, such as myocardial ischemia, dilated cardiomyopathy, or valvular heart disease [4]. In patients with symptomatic mitral regurgitation (MR), previous studies evaluating the effect of surgical or transcatheter edge-to-edge mitral valve repair (TEER) are contradicting. While surgery has been shown to effectively reduce SDB and eliminate CSA in individual patients [5–7], a small case series of patients undergoing MitraClip implantation did not detect a reduction of SDB severity [8]. With another study demonstrating modest SDB amelioration after TEER in a population largely suffering from OSA [9], the value of minimal-invasive MR therapy in reducing CSA, particularly in comparison to surgery, remains to be established. Therefore, the aim of the present study was to investigate the effect of TEER on CSA and elucidate potential mechanisms associated with SDB attenuation.

## Methods

### Study population

**Fig. 1:**
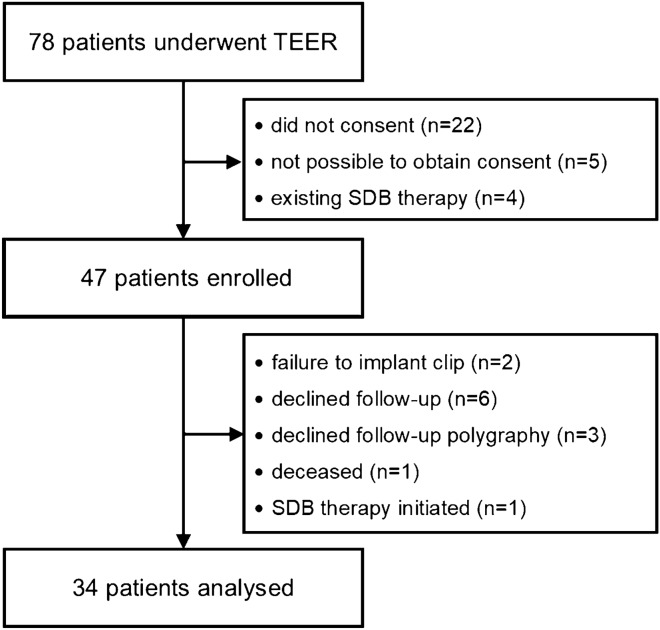
Study flow chart. *SDB*, sleep-disordered breathing; *TEER*, transcatheter edge-to-edge mitral valve repair

From October 2019 to February 2021, we enrolled consecutive patients in a prospective observational study at the University Heart Center Regensburg (Fig. 1). Inclusion criterion was symptomatic moderate-to-severe or severe MR treated with TEER. Patients with previously established specific SDB therapy were excluded. The decision for TEER in each patient was made by an interdisciplinary Heart Team consisting of an interventional cardiologist, a cardiac surgeon, and an anaesthesiologist. Participants with failure to implant the device, incomplete follow-up, or initiation of specific SDB therapy before follow-up were excluded from analysis.

### Study design

Baseline respiratory polygraphy was performed during the hospital stay in the night before TEER. Patients underwent prior cardiac recompensation therapy, if needed. Follow-up recordings were acquired four weeks after TEER in an outpatient setting, instructing participants to use the device for one night at home. Additionally, all participants underwent comprehensive clinical and echocardiographic assessment at baseline and four weeks after TEER, including New York Heart Association (NYHA) functional class, six-minute walk distance, and laboratory measurements of natriuretic peptides. Patient-reported quality of life was evaluated using the EuroQol EQ-5D-5L questionnaire; results are reported as EQ-5D index value (from 0 [worst] to 1 [best]) and EQ visual analogue scale (from 0 [worst] to 100 [best]). MR grading was based on colour and continuous wave Doppler examination in accordance with current guidelines [10, 11]. Regurgitation grade was scored from I to IV (I: mild, II: mild-to-moderate, III: moderate-to-severe, IV: severe). Stroke volume was estimated noninvasively by measuring doppler-derived velocity time integral of the left ventricular outflow tract. TEER was performed by experienced interventionalists using the MitraClip (Abbott Vascular, Menlo Park, USA) or PASCAL system (Edwards Lifesciences, Irvine, USA) under general anaesthesia, guidance by fluoroscopy, and three-dimensional transoesophageal echocardiography.

### Respiratory polygraphy

Nocturnal respiratory polygraphy was performed using a three-channel SDB-monitoring device (ApneaLink plus, ResMed, USA), recording nasal flow, thoracic effort, and pulse oximetry. Performance of the device with automatic scoring in patients without known heart disease was validated in several studies, reporting a sensitivity of 73–94% and a specificity of 85–95% using an apnoea-hypopnoea index (AHI) cutoff value of 15/h [12–14]. A previous study on the use of the SDB-monitoring device in patients with chronic HF reported a sensitivity of 73% and a specificity of 87% for detecting SDB when compared with the gold standard polysomnography [1]. Recordings were analysed and scored by trained medical staff. The default settings of the device were used for the definitions of apnoea, hypopnoea, and desaturation: apnoea was defined as a ≥ 80% decrease in airflow for ≥ 10 s, hypopnoea as a decrease in airflow by ≥ 50–80% for ≥ 10 s, and desaturation as a ≥ 4% decrease in oxygen saturation. If > 50% of apnoeas were obstructive, patients were classified as OSA. Otherwise, patients were classified as CSA. CSR was defined as ≥ 3 episodes of continuous cycles of waxing and waning tidal volumes with periods of hyperventilation separated by apnoea/hypopnoeas. A prior validation study on CSR detection in both HF and non-HF patients reported a sensitivity of 87% and specificity of 95% for the device [15]. AHI cut-off values for mild and moderate-to-severe SDB were defined as ≥ 5/h and ≥ 15/h, respectively.

### Statistical analysis

Continuous variables are reported as mean ± standard deviation when normally distributed, continuous variables with skewed distribution as median with interquartile range [first quartile-third quartile]. Categorical variables are presented in numbers and percentages. Differences in unpaired data were assessed using students t-test for normally distributed data, Mann–Whitney-*U* test for ordinal or skewed data, Pearson’s chi-squared test for nominal data, and Fisher’s exact test for dichotomous data. Differences in paired samples were tested using paired *t* tests for normally distributed data, Wilcoxon signed-rank tests for skewed or ordinal data, and McNemar’s test for dichotomous data. To identify predictors of SDB and CSR improvement, binary logistic regression models were calculated. Predictors with *p* < 0.05 in univariate analysis were included in the multivariate analysis. A two-sided *p* value of < 0.05 was considered statistically significant. All statistical analyses were performed using SPSS Statistics 26 (IBM, Armonk, USA).

## Results

### Baseline characteristics and procedural outcome

Of 78 patients who underwent TEER from October 2019 to February 2021, 47 patients were enrolled. A total of 34 patients completed the follow-up and were available for analysis (Fig. 1). Mean age of the study population was 72 ± 12 years, and 14 (41%) patients were of female gender. Most patients presented with MR grade IV (74%) of secondary or mixed aetiology (79%). Reduced left ventricular ejection fraction (< 50%) was present in 23 (68%) participants. With 26 (74%) patients exhibiting NYHA functional class III or worse, the study population showed clinical signs of advanced HF.

Baseline characteristics in relation to the presence of SDB are shown in Table 1*.* Patients with moderate-to-severe SDB (AHI ≥ 15/h) had worse functional status at baseline in terms of NYHA class (III or IV 90% vs. 53%, *p* = 0.036). Also, SDB was associated with more severe tricuspid regurgitation (severe in 63% vs. 20%, *p* = 0.047) and worse quality of life (EQ visual analogue scale 50 [40–55] vs. 60 [50–70], *p* = 0.045). No differences were detected in MR severity, left ventricular ejection fraction, or comorbidities. Yet, we noted a trend towards patients with moderate-to-severe SDB being more frequently male (74% vs. 40%, *p* = 0.080) and having higher systolic pulmonary artery pressure (sPAP, 46 ± 15 vs. 37 ± 11 mmHg, *p* = 0.086).

**Table 1:** Clinical and echocardiographic characteristics at baseline, stratified by the presence of moderate-to-severe SDB (AHI ≥ 15/h)

	Moderate-to-severe SDB (AHI ≥ 15/h)		*p* value
	Yes (*n* = 19)	No (*n* = 15)	
Age, years	74 ± 12	70 ± 12	0.320
Female gender	5 (26%)	9 (60%)	0.080
BMI, kg/m^2^	25.6 ± 3.7	25.6 ± 4.3	0.972
Coronary artery disease	14 (74%)	9 (60%)	0.475
Atrial fibrillation	14 (73%)	8 (53%)	0.288
Diabetes mellitus	3 (16%)	5 (33%)	0.417
EuroSCORE II, %	4.2 [2.1–9.4]	4.3 [2.6–10.5]	0.813
GFR, ml/min	48 ± 22	54 ± 22	0.394
NTproBNP, pg/ml	2211 [759–4933]	2148 [810–2793]	0.910
NYHA functional class			
I	0	1 (7%)	**0.036**
II	2 (11%)	6 (40%)	
III	15 (79%)	8 (53%)	
IV	2 (11%)	0	
Six-minute walk distance, m	229 ± 102	250 ± 80	0.580
Echocardiography			
MR of secondary or mixed aetiology	15 (79%)	12 (80%)	1.000
MR grade			
III	4 (21%)	5 (33%)	0.462
IV	15 (79%)	10 (67%)	
PISA EROA, cm^2^	0.31 ± 0.09	0.31 ± 0.12	0.935
LVEF, %	44 ± 13	40 ± 15	0.366
Stroke volume (LVOT), ml	51 ± 15	48 ± 13	0.554
Left ventricular end diastolic volume, ml	168 ± 62	191 ± 87	0.376
Left atrial volume index, ml/m^2^	91 ± 57	71 ± 20	0.207
Tricuspid regurgitation grade			
Mild	3 (16%)	4 (27%)	**0.047**
Moderate	4 (21%)	8 (53%)	
Severe	12 (63%)	3 (20%)	
sPAP, mmHg	46 ± 15	37 ± 11	0.086
Quality of life			
EQ-5D index^a^	0.74 [0.57–0.86]	0.83 [0.73–0.92]	0.053
EQ visual analogue scale^b^	50 [40–55]	60 [50–70]	**0.045**

Variables are expressed as *n* (%), mean ± standard deviation, or median [interquartile range], as appropriate

*AHI*, Apnoea-Hypopnoea Index; *BMI*, body mass index; *EROA*, effective regurgitation orifice area; GFR, glomerular filtration rate; *LVEF*, left ventricular ejection fraction; *LVOT*, left ventricular outflow tract; *MR*, mitral regurgitation; *NYHA*, New York Heart Association; *PISA*, proximal isovelocity surface area; SDB, sleep-disordered breathing; *sPAP*, systolic pulmonary artery pressure

^a^From 0 (worst) to 1 (best)

^b^From 0 (worst) to 100 (best)

Procedural details of TEER are shown in Supplementary Table 1. SDB was not associated with an increase in periinterventional ventilation time or length of hospital stay. In the follow-up examination four weeks after the procedure, reduction of MR to grade I or II was achieved in 29 (85%) patients. Concomitantly, echocardiography revealed a reduction of left atrial volume (82 ± 45 vs. 71 ± 41 ml/m^2^, *p* < 0.001) and systolic pulmonary artery pressure (42 ± 14 vs. 37 ± 14 mmHg, *p* = 0.003). Patients showed significant improvement in both NYHA functional status (class III or IV 74% vs. 15%, *p* < 0.001) and six-minute walk distance (252 ± 92 vs 295 ± 104 m, *p* = 0.002). This was accompanied by an increase in patient-reported quality of life (EQ-5D index 0.80 [0.66–0.87] vs. 0.88 [0.66–0.94], *p* = 0.032). Of note, intake of HF medication or diuretics and body weight did not change from baseline to the four weeks follow-up (Supplementary Table 2).

### Respiratory parameters

**Fig. 2:**
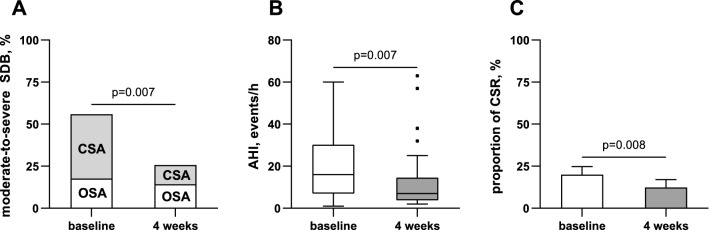
Prevalence of moderate-to-severe SDB (**A**), Apnoea-Hypopnoea index (**B**) and proportion of Cheyne-Stokes respiration (**C**) at baseline and four weeks after TEER. **B** Data are shown as tukey-style box plots. **C** Data are shown as mean ± SEM. *AHI*, Apnoea-Hypopnoea index; *CSA*, central sleep apnoea; *CSR*, Cheyne-Stokes respiration; *OSA*, obstructive sleep apnoea; *SDB*, sleep-disordered breathing

Results of respiratory polygraphy of the whole study population are shown in Table 2*.* At baseline, 19 (56%) patients showed moderate-to-severe SDB (AHI ≥ 15/h), of whom 13 (68%) had CSA (Fig. 2). In the follow-up examination 4 weeks after TEER, the number of patients with moderate-to-severe SDB was reduced by more than half to 9 (24%, *p* = 0.007). Alongside, AHI decreased substantially from a median of 16 [7–30] to 7 [4–15]/h (*p* = 0.007). This reduction was driven by a decrease of central events: central apnoea index significantly decreased by a median of 75% from baseline to follow-up (*p* = 0.023). Similarly, proportion of recorded time spent in CSR significantly decreased from 6 [0–34] to 0 [0–8] % (*p* = 0.008). As a consequence, CSA resolved in 9 of the 13 affected patients (*p* = 0.022). In contrast, the frequency of obstructive apnoeas remained similar (*p* = 0.153).

**Table 2:** Respiratory polygraphy at baseline and four weeks after TEER

	Baseline	4 weeks after TEER	*p* value
Moderate-to-severe SDB (AHI ≥ 15/h)	19 (56%)	8 (24%)	**0.007**
OSA	6 (18%)	5 (15%)	1.000
CSA	13 (38%)	4 (12%)	**0.022**
AHI, events/h	16 [7–30]	7 [4–15]	**0.007**
Apnoea index, events/h	6 [2–11]	2 [1–6]	**0.004**
Obstructive apnoea index, events/h	1 [1–3]	1 [0–3]	0.153
Central apnoea index, events/h	2 [0–9]	1 [0–3]	**0.023**
Hypopnoea index, events/h	6 [3–11]	3 [2–6]	**0.038**
Proportion of Cheyne-Stokes respiration, %	6 [0–34]	0 [0–8]	**0.008**
Oxygen desaturation index, events/h	15 [8–32]	8 [6–16]	**0.015**
Mean SpO_2_, %	92 ± 2	92 ± 2	0.965
Minimum SpO_2_, %	78 ± 8	78 ± 7	0.926

Variables are expressed as *n* (%), mean ± standard deviation or median [interquartile range], as appropriate

*AHI*, Apnoea-Hypopnoea index; *CSA*, central sleep apnoea; *OSA*, obstructive sleep apnoea; *SpO*_*2*_, peripheral oxygen saturation; *SDB*, sleep-disordered breathing; *TEER*, transcatheter edge-to-edge mitral valve repair

Additionally, we performed a subanalysis of patients with at least mild SDB (AHI ≥ 5/h; Supplementary Table 3). Similar to above findings, AHI, central apnoea events, and CSR significantly decreased from baseline to four weeks after TEER. Yet, we also observed a trend towards a reduction of obstructive apnoeas from baseline to follow-up (2 [1–5] vs. 1 [0–3]/h, *p* = 0.056).

### Predictors of reduction of sleep-disordered breathing

**Fig. 3:**
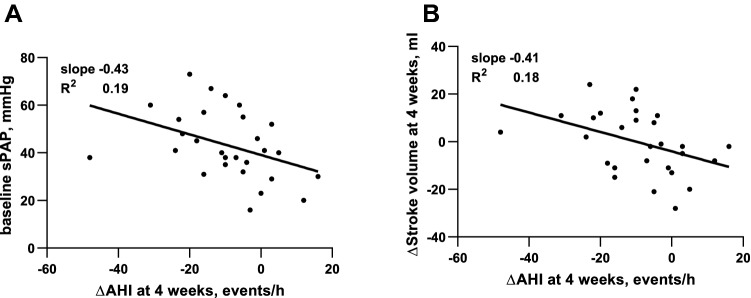
Correlation between AHI reduction four weeks after TEER and baseline systolic pulmonary artery pressure (**A**) or stroke volume increase (**B**) in patients with at least mild SDB (AHI ≥ 5/h, *n* = 28). Data are shown as scatter plots with linear regression lines. *AHI*, Apnoea-Hypopnoea index; *sPAP*, systolic pulmonary artery pressure

To identify predictors of significant AHI reduction four weeks after TEER, univariate and multivariate logistic binary regression was conducted in patients with at least mild SDB at baseline (Table 3). Successful reduction of AHI was defined as a > 50% decrease from baseline to follow-up. Both increase in cardiac stroke volume (odds ratio [OR] per ml 1.26 [1.07–1.49], *p* = 0.006) and higher sPAP at baseline (OR per mmHg 1.11 [1.02–1.20], *p* = 0.016) were predictors of successful AHI reduction (Fig. 3). In multivariate analysis, increase in stroke volume remained an independent predictor (OR per ml 1.24 [1.05–1.48], *p* = 0.013). Nor echocardiographic reduction of MR grade or change in serum NTproBNP levels were associated with AHI reduction. Also, gender, left ventricular function, MR of secondary aetiology, or cardiac comorbidities were not predictive of an improvement of SDB. To confirm our results, we additionally evaluated predictors of successful reduction of CSR, defined as a > 50% decrease from baseline to follow-up (Supplementary Table 4). Congruous to above findings, higher sPAP at baseline and increase in stroke volume were predictive of CSR reduction in univariate analysis (OR per mmHg 1.14 [1.00–1.31], *p* = 0.046; OR per ml 1.17 [1.01–1.34], *p* = 0.034).

**Table 3:** Predictors of significant AHI reduction (> 50%) four weeks after TEER in patients with at least mild SDB (AHI ≥ 5/h, *n* = 28)

Variable	Univariate analysis		Multivariate analysis^a^	
	Odds ratio (95% CI)	*p* value	Odds ratio (95% CI)	*p* value
Male gender	0.67 (0.14–3.19)	0.612		
Age, years	1.07 (0.99–1.15)	0.087		
Coronary artery disease	0.58 (0.12–2.88)	0.507		
Diabetes mellitus	0.17 (0.02–1.67)	0.128		
Atrial fibrillation	0.42 (0.09–2.06)	0.288		
MR of secondary or mixed aetiology	1.21 (0.22–6.80)	0.827		
NYHA class at baseline	2.46 (0.63–9.62)	0.195		
log(NTproBNP) at baseline, pg/ml	1.31 (0.69–2.52)	0.412		
∆log(NTproBNP) at four weeks, pg/ml	1.78 (0.52–6.06)	0.356		
LVEF at baseline, %	1.01 (0.96–1.07)	0.662		
sPAP at baseline, mmHg	1.11 (1.02–1.20)	**0.016**	1.09 (0.97–1.21)	0.147
∆MR grade at four weeks	1.49 (0.55–4.17)	0.435		
∆Stroke volume at four weeks, ml	1.26 (1.07–1.49)	**0.006**	1.24 (1.05–1.48)	**0.013**

*AHI*, Apnoea-Hypopnoea index; *BMI*, body mass index; *CI*, confidence interval; *LVEF*, left ventricular ejection fraction; *MR*, mitral regurgitation; *NYHA*, New York Heart Association; *sPAP*, systolic pulmonary artery pressure; *TEER*, transcatheter edge-to-edge mitral valve repair

^a^Variables with *p* < 0.05 in univariate analysis were included in multivariate analysis

### Clinical outcome in patients with central sleep apnoea

Considering the observed strong effects of TEER on central respiratory events, we conducted a subanalysis of clinical and echocardiographic outcomes in patients with CSA (*n* = 13; Supplementary Table 5). Mirroring the results of the whole study population, the procedure was associated with a strong reduction of MR grade (III or IV 100% vs. 16%, *p* < 0.001) and improvement in NYHA functional status (class III or IV 84% vs 31%, *p* = 0.001) at the four weeks follow-up. Whereas left ventricular parameters and serum NTproBNP levels remained unchanged, left atrial reverse remodeling was observed (left atrial volume index 71 ± 26 vs. 64 ± 21 ml/m^2^, *p* = 0.030). Also, patients reported a pronounced increase in quality of life four weeks after the procedure (EQ-5D index 0.65 [0.43–0.76] vs. 0.80 [0.54–0.95], *p* = 0.004).

## Discussion

Our study provides systematic investigation on SDB in patients treated with minimal-invasive mitral valve repair in a high-risk population with symptomatic MR. As the main finding, TEER was associated with a significant reduction in central sleep apnoea and hypopnoea events in the short term, resulting in the resolution of moderate-to-severe SDB in approximately half of the affected patients. Likewise, proportion of CSR, a hallmark of advanced HF [3], substantially decreased after treatment of MR. Furthermore, we identified elevated baseline sPAP and increase in cardiac stroke volume as independent predictors of AHI and CSR reduction after TEER.

### Therapy of mitral regurgitation ameliorates central sleep apnoea and Cheyne-Stokes respiration

Previous data on the effects of MR therapy on SDB and its subtype CSA are limited. In two case reports, nearly complete resolution of CSA was observed after surgical mitral valve therapy, accompanied by a reduction in circulation delay [6, 7]. In a systematic investigation of 82 patients, mitral valve surgery was associated with a significant reduction in AHI three months postoperatively [5]. Yet, due to the lack of respiratory effort measurements, the effect on SDB subtypes remained unclear. In this context, our study adds two important aspects to current knowledge on the impact of MR therapy on SDB: First, the effect of mitral valve surgery on SDB was reproduced using transcatheter repair, both confirming previous results and demonstrating the equivalence of TEER to surgical therapy with a view to improving central sleep apnoea. Second, our findings confirm that the effect of successful mitral valve therapy on SDB is strongly connected with a reduction of CSA and CSR.

Two previous studies investigated the course of SDB in patients undergoing TEER. Contrary to our findings, Spiesshoefer et al. did detect a reduction in respiratory event lengths, but not in SDB severity in a study of 20 patients undergoing MitraClip implantation [8]. With baseline characteristics, MR severity, SDB prevalence, and subtype distribution being comparable to our study population, we may only speculate about the reasons for this discrepancy. While follow-up polygraphy was performed 4 weeks after TEER in our protocol, Spiesshoefer et al. conducted the second polygraphy only days after the intervention. As such, their findings might have been confounded by postoperative fluid overload and/or anaesthesia. More importantly, it has been shown that restoration of impaired chemoreceptor sensitivity after therapeutic intervention takes 2–6 weeks to set in [16]. Likewise, cardiac reverse remodeling and its systemic effects after successful TEER might manifest after a certain delay. In summary, we consider it likely that the follow-up polygraphy in Spiesshoefer et. al was carried out too early to evaluate the effect. Still, the observed decrease of respiratory event lengths is well in line with our results and may constitute a precursor to the remarkable effect on SDB severity in our study.

In another study, Daher et al. investigated the effect of TEER on SDB in a population with baseline characteristics similar to our study [9]. They observed less pronounced results, with AHI reduction only being significant in a subgroup of patients with moderate-to-severe SDB. The vast majority of patients in Daher et al. showed predominant OSA, with only four patients suffering from CSA and only two exhibiting significant CSR. This is distinct from the phenotype found in our study and in large registry studies on SDB in chronic HF, which reported predominant CSA in 31% and periodic breathing in 41% of patients [3, 17]. Consequently, the results from the previous study might more adequately reflect the effect of TEER on OSA, whereas our study adds insight on the course of SDB in a population with high CSA burden. As we observed that the reduction in apnoeas was driven by a decrease in central events, it is likely that the larger proportion of patients with CSA led to stronger effects on SDB severity in our study.

### Reduction of central sleep apnoea after transcatheter mitral valve repair is associated with increased cardiac stroke volume and pulmonary decongestion

In our study, high baseline sPAP and increase in cardiac stroke volume after TEER were predictive of both AHI and CSR reduction. Considering the pathophysiology of SDB in HF, these findings offer conclusive mechanistic explanations for the observed effect of TEER. CSA is characterized by a dysregulation of the feedback loop between chemoreceptors and respiratory effort [18]. The hemodynamic consequences of HF promote CSA by compromising two components of the feedback loop: reduced cardiac output increases circulatory delay between lungs and chemoreceptors, perpetuating the cycle of periodic ventilatory over- and undershoot which results in CSR [19]. Second, elevated left ventricular filling pressure and left atrial distension increase chemoreceptor sensitivity, aggravating the chemoreflex response to fluctuations in blood oxygen and carbon dioxide levels (loop gain) initiating the Cheyne-Stokes cycle [20, 21].

Our results indicate that the hemodynamic effects of TEER target both components of the feedback loop: an increase in cardiac stroke volume, which was shown to predict SDB reduction, improves cardiac output, reducing circulatory delay. Additionally, as observed in our study, sPAP and left atrial distension decrease after TEER, which reduces loop gain by lowering chemoreceptor sensitivity. In accordance with this consideration, we found that high sPAP at baseline is predictive of SDB reduction after TEER, identifying patients with high left ventricular filling pressure who profit from pulmonary decongestion. Apart from these mechanisms, nocturnal fluid shifts from the legs into neck and lungs occurring in HF have been shown to promote upper airway narrowing and hyperventilation, triggering both OSA and CSA in a unifying pathophysiological concept [22]. Hence, amelioration of right-heart failure by successful therapy of MR may have contributed to the observed effect on SDB.

### Strengths and limitations

Our study provides insight on the course of SDB in a well-characterized group of high-risk patients with symptomatic MR, reflecting the population treated with TEER in contemporary clinical care. By combining comprehensive non-invasive cardiac evaluation and polygraphy, we were able to elucidate hemodynamic changes associated with SDB improvement. As the baseline polygraphy was performed in the night before TEER, it was ensured that the patients were in a compensated condition during the measurement. This conclusion is strengthened by the fact that the participants did not show weight loss from baseline to the four weeks follow-up. In addition, neither HF medication nor diuretics changed from baseline to the four weeks follow-up. Considering that both fluid overload and escalation of HF medication have been shown to attenuate SDB [23–25], the absence of these confounders support the validity of the main findings of our study.

Our study might have several limitations. Like previous investigations on SDB in mitral valve disease, our study lacks a control group. Therefore, we cannot rule out that unknown confounders influenced the results. To enable SDB diagnostics timely before TEER and in an outpatient setting for follow-up, a portable three-channel device was used instead of polysomnography. While the latter is the diagnostic gold standard, portable devices have been validated in multiple studies and have shown adequate diagnostic accuracy in patients with chronic HF [1]. The limited sample size may have restricted statistical power. In particular, results of multivariate analysis may be considered exploratory and should be validated in a larger cohort.

Subjective sleepiness is a key feature of SDB in patients without HF, guiding both diagnosis and therapy. However, the opposite is true for HF patients with sleep apnoea: this population is characterized by the remarkable absence of sleepiness symptoms despite objective evidence of reduced sleep quality [26]. Multiple observational studies consistently failed to demonstrate differences in patient-reported daytime sleepiness in HF patients with or without SDB, encompassing both CSA and OSA [27–29]. Hence, we refrained from assessing subjective sleepiness in our patients, as we deemed it unhelpful in giving additional insight into the relationship between SDB and TEER. This consideration is strengthened by the results of Daher et al., who did not find differences in self-reported sleepiness in patients with or without sleep apnoea undergoing TEER [9].

### Clinical implications

SDB is a very common comorbidity in patients with chronic HF and associated with worse prognosis [1, 2]. While treatment of OSA by positive airway pressure may improve quality of life in subjects with HF, the therapeutic approach to CSA, which is more common in these patients, remains challenging [4]. Although adaptive servo ventilation effectively reduces CSA, it counter-intuitively increases mortality in patients with reduced left ventricular ejection fraction [30]. This detrimental effect was shown to be even more pronounced in patients with a high proportion of CSR. These results foster the concept of CSA being a compensatory mechanism with protective effects in severe HF [31], which in consequence must not be suppressed by ventilation therapy. Instead, given its strong prognostic burden [32], the presence of CSA may serve as an indicator of advanced disease which warrants intensified guideline-based therapy of the underlying heart condition by HF medication, cardiac resynchronization, and TEER. In line with this consideration, SDB was associated with worse functional status, quality of life, and more severe tricuspid regurgitation in our study. Noteworthy, tricuspid regurgitation itself has been demonstrated to predict poor outcome in HF patients in general [33, 34] as well in those with MR in particular [35], corroborating the link between CSA and advanced HF.

Conversely, attenuation of CSA may be a surrogate marker for successful HF therapy. In this context, the observed amelioration of CSA after TEER in our study may imply a significant reduction of total cardiovascular risk, mirroring the results of the COAPT study on mortality and morbidity [36]. Indeed, CSA patients showed favourable clinical outcomes in our study in terms of MR reduction, functional status, and quality of life, substantiating the relationship between SDB and cardiac function. Of note, while SDB has been shown to predict pulmonary complications after cardiac surgery [37], it was not associated with increased ventilation time or adverse events in our study, confirming the good safety profile the procedure. As a reduction in CSA was also observed after well-established therapies such as ACE inhibition [23] and cardiac resynchronization [38], our data underline the value of TEER being an effective therapy for advanced HF.

## Conclusion

Predominant central SDB is very common in high-risk patients with MR. Reduction of MR by TEER is associated with a strong improvement in SDB severity, primarily driven by a decrease of central events and CSR. Increase in cardiac stroke volume and high pulmonary artery pressure at baseline predict SDB improvement and CSR resolution, suggesting decrease of circulatory delay and pulmonary decongestion as the underlying mechanisms. Considering the strong prognostic burden of CSA, our results underline that TEER is an effective treatment option in advanced HF.

## Supplementary Information

Below is the link to the electronic supplementary material.

Supplementary file1 (PDF 361 kb)

## Data Availability

The data that support the findings of this study are available from the corresponding author upon request.
