# Supplementary material for: Impact of transcatheter edge-to-edge mitral valve repair on central sleep apnoea
Source: Clin Res Cardiol. 2022 Dec 12;112(5):594–604. doi: 10.1007/s00392-022-02139-3 (PMC10160214; doi:10.1007/s00392-022-02139-3)
Supplement: Supplementary file 1 — Supplementary file1 (PDF 361 kb) [file 392_2022_2139_MOESM1_ESM.pdf]

Impact of transcatheter edge-to-edge mitral valve repair on central sleep apnoea

Michael G. Paulus, Tobias Liedtke, Michael Hamerle, Christian Schach, Lars S. Maier, Stefan Stadler, Christoph Birner, Kurt Debl, Michael Arzt, Bernhard Unsöld, Christine Meindl

Clinical Research in Cardiology

Supplementary Information

Corresponding author:
Michael G. Paulus, MD
Department of Internal Medicine II, University Hospital Regensburg, Germany
E-mail: michael.paulus@ukr.de

Supplementary Tables

Supplementary Table 1: Procedural details of TEER in the study population (n=34).

	Moderate-to-severe SDB (AHI ≥15/h)		p-value
	**Yes (n=19)**	**No (n=15)**	
**No. of implanted devices**			
1	7 (37%)	9 (60%)	0.215
2	11 (58%)	6 (40%)	
3	1 (5%)	0	
**Device type**			
MitraClip	13 (87%)	16 (84%)	1.000
PASCAL	2 (13%)	3 (16%)	
**Length of hospital stay, days**	6 [4-9]	8 [5-11]	0.622
**Ventilation time, hours**	2.75 [2.25-3.50]	2.75 [2.25-3.00]	0.929
**Adverse events**			
Leaflet detachment	2 (11%)	0	0.492
Urgent heart surgery	0	0	
Myocardial infarction	0	0	
Stroke	0	0	
Major bleeding	1 (5%)	0	1.000
Pericardial tamponade	0	0	
Mechanical ventilation >48h	0	0	
Shock	0	0	
Death	0	0	

Variables are expressed as n (%) or median [interquartile range], as appropriate.
AHI, Apnoea-Hypopnoea Index; SDB, sleep-disordered breathing; TEER, transcatheter edge-to-edge mitral valve repair.

Supplementary Table 2: Clinical and echocardiographic results four weeks after TEER.

	Baseline	4 weeks after TEER	p-value
**NYHA functional class**			
I	1 (3%)	3 (9%)	**<0.001**
II	8 (24%)	20 (59%)	
III	23 (68%)	11 (32%)	
IV	2 (6%)	0	
**Six-minute walk distance, m**	252±92	295±104	**0.002**
**NTproBNP, pg/ml**	2180 [797-3391]	2261 [1294-4239]	0.873
**Weight, kg**	73.6±11.3	74.9±11.7	0.271
**Echocardiography**			
**MR grade**			
I	0	20 (59%)	**<0.001**
II	0	9 (26%)	
III	9 (26%)	4 (12%)	
IV	25 (74%)	1 (3%)	
**Tricuspid regurgitation grade**			
Mild	7 (21%)	14 (41%)	**0.005**
Moderate	12 (35%)	11 (32%)	
Severe	15 (44%)	9 (26%)	
**LVEF, %**	42±14	42±14	0.942
**Stroke volume (LVOT), ml**	49±14	48±16	0.652
**Left ventricular end diastolic volume, ml**	178±74	168±81	0.339
**Left atrial volume index, ml/m²**	82±45	71±41	**<0.001**
**sPAP, mmHg**	42±14	37±14	**0.003**
**TAPSE, mm**	18±4	19±4	**0.047**
**MV mean pressure gradient, mmHg**	2.2±0.9	3.4±1.3	**0.002**
**Medication**			
**ACE inhibitor/AT$_1$ antagonist**	22 (65%)	19 (56%)	0.375
Dose[a], %	31 [25-50]	25 [25-50]	0.671
**ARNI**	6 (18%)	8 (24%)	0.625
Dose[a], %	50 [25-63]	38 [25-50]	0.374
**Beta blocker**	31 (91%)	29 (85%)	0.625
Dose[a], %	50 [25-75]	50 [25-88]	0.660
**Aldosterone antagonist**	20 (59%)	20 (59%)	1.000
Dose[a], %	50 [50-100]	50 [50-100]	0.834
**Loop diuretic**	31 (91%)	32 (94%)	1.000
Dose, mg furosemide equivalent[b]	40 [20-60]	40 [40-68]	0.923
**Quality of life**			
**EQ-5D index[c]**	0.80 [0.66-0.87]	0.88 [0.66-0.94]	**0.032**
**EQ visual analogue scale[d]**	53 [50-66]	65 [50-76]	0.089

[a] Percentage of target dose recommended by current heart failure guidelines [1] [b] 10 mg torasemide was converted to 20 mg furosemide equivalent [c] from 0 (worst) to 1 (best) [d] from 0 (worst) to 100 (best)
Variables are expressed as n (%), mean±standard deviation, or median [interquartile range], as appropriate.
ACE, Angiotensin-converting-enzyme; ARNI, Angiotensin receptor neprilysin inhibitor; AT$_1$, Angiotensin II receptor type 1; LVEF, left ventricular ejection fraction; LVOT, left ventricular outflow tract; MR, mitral regurgitation; MV, mitral valve; NYHA, New York Heart Association; sPAP, systolic pulmonary artery pressure; TAPSE, tricuspid annular plane systolic excursion; TEER, transcatheter edge-to-edge mitral valve repair.

Supplementary Table 3: Subanalysis of respiratory polygraphy in patients with at least mild SDB (AHI ≥5/h, n=28).

	Baseline	4 weeks after TEER	p-value
**AHI, events/h**	17 [9-37]	7 [4-17]	**0.001**
**Apnoea index, events/h**	3 [7-14]	2 [1-6]	**<0.001**
Obstructive apnoea index, events/h	2 [1-5]	1 [0-3]	0.056
Central apnoea index, events/h	2 [1-13]	1 [0-2]	**0.004**
**Hypopnoea index, events/h**	8 [5-13]	3 [2-6]	**0.018**
**Proportion of Cheyne-Stokes respiration, %**	10 [3-40]	0 [0-12]	**0.009**
**Oxygen desaturation index, events/h**	18 [12-36]	9 [5-21]	**0.003**
**Mean SpO_2, %**	91 [90-93]	92 [90-93]	0.846
**Minimum SpO_2, %**	79 [76-82]	78 [76-83]	0.731

Variables are expressed as median [interquartile range].
AHI, Apnoea-Hypopnoea Index; SDB, sleep-disordered breathing; SpO_2, peripheral oxygen saturation; TEER, transcatheter edge-to-edge mitral valve repair.

Supplementary Table 4: Predictors of significant CSR reduction (>50%) four weeks after TEER in patients with >5% CSR at baseline (n=21).

	Univariate analysis		Multivariate analysis[a]	
	**Odds ratio (95% CI)**	**p-value**	**Odds ratio (95% CI)**	**p-value**
**Male gender**	0.30 (0.03-3.25)	0.322		
**Age, years**	1.04 (0.96-1.13)	0.366		
**Coronary artery disease**	1.38 (0.18-10.65)	0.760		
**Atrial fibrillation**	8.00 (0.96-66.45)	0.054		
**MR of secondary or mixed aetiology**	2.00 (0.24-16.61)	0.521		
**NYHA class at baseline**	1.56 (0.38-6.40)	0.537		
**log(NTproBNP) at baseline, pg/ml**	0.69 (0.24-2.00)	0.489		
**∆log(NTproBNP) at four weeks, pg/ml**	2.00 (0.37-10.86)	0.424		
**LVEF at baseline, %**	1.03 (0.96-1.10)	0.443		
**sPAP at baseline, mmHg**	1.14 (1.00-1.31)	**0.046**	1.14 (0.98-1.32)	0.080
**∆MR grade at four weeks**	1.00 (0.29-3.50)	1.000		
**∆Stroke volume at four weeks, ml**	1.17 (1.01-1.34)	**0.034**	1.18 (0.98-1.41)	0.075

[a] Variables with p<0.05 in univariate analysis were included in multivariate analysis.
BMI, body mass index; CI, confidence interval; CSR, Cheyne-Stokes respiration; LVEF, left ventricular ejection fraction; MR, mitral regurgitation; NYHA, New York Heart Association; sPAP, systolic pulmonary artery pressure; TEER, transcatheter edge-to-edge mitral valve repair.

Supplementary Table 5: Subanalysis of clinical and echocardiographic results in patients with CSA (n=13).

	Baseline	4 weeks after TEER	p-value
**NYHA functional class**			
I	0	1 (8%)	**0.001**
II	2 (15%)	8 (62%)	
III	9 (69%)	4 (31%)	
IV	2 (15%)	0	
**Six-minute walk distance, m**	252±121	283±140	0.237
**NTproBNP, pg/ml**	2450 [1401-6496]	3036 [2261-7501]	0.388
**Weight, kg**	75.4±13.0	75.5±12.0	0.937
**Echocardiography**			
**MR grade**			
I	0	6 (46%)	**<0.001**
II	0	5 (38%)	
III	2 (15%)	1 (8%)	
IV	11 (85%)	1 (8%)	
**Tricuspid regurgitation grade**			
Mild	2 (15%)	3 (23%)	0.109
Moderate	3 (23%)	6 (46%)	
Severe	8 (62%)	4 (31%)	
**LVEF, %**	41±14	39±13	0.103
**Stroke volume (LVOT), ml**	45±13	47±18	0.734
**Left ventricular end diastolic volume, ml**	164±56	162±80	0.899
**Left atrial volume index, ml/m²**	71±26	64±21	**0.030**
**sPAP, mmHg**	45±15	43±18	0.745
**TAPSE, mm**	18±4	18±3	0.894
**MV mean pressure gradient, mmHg**	1.8±0.7	2.8±0.7	**0.018**
**Quality of life**			
**EQ-5D index[a]**	0.65 [0.43-0.76]	0.80 [0.54-0.95]	**0.004**
**EQ visual analogue scale[b]**	50 [35-58]	70 [45-75]	**0.001**

[a] from 0 (worst) to 1 (best) [b] from 0 (worst) to 100 (best)
Variables are expressed as n (%), mean±standard deviation, or median [interquartile range], as appropriate.
CSA, central sleep apnoea; LVEF, left ventricular ejection fraction; LVOT, left ventricular outflow tract; MR, mitral regurgitation; MV, mitral valve; NYHA, New York Heart Association; sPAP, systolic pulmonary artery pressure; TAPSE, tricuspid annular plane systolic excursion.

References

1. McDonagh TA, Metra M, Adamo M, Gardner RS, Baumbach A, Böhm M, Burri H, Butler J, Čelutkienė J, Chioncel O, Cleland JGF, Coats AJS, Crespo-Leiro MG, Farmakis D, Gilard M, Heymans S, Hoes AW, Jaarsma T, Jankowska EA, Lainscak M, Lam CSP, Lyon AR, McMurray JJV, Mebazaa A, Mindham R, Muneretto C, Francesco Piepoli M, Price S, Rosano GMC, Ruschitzka F, Kathrine Skibelund A (2021) 2021 ESC Guidelines for the diagnosis and treatment of acute and chronic heart failure. European Heart Journal. doi: 10.1093/eurheartj/ehab368
